# Assembly and Annotation of the Tetraploid *Salsola tragus* (Russian Thistle) Genome

**DOI:** 10.1093/gbe/evaf014

**Published:** 2025-01-27

**Authors:** John M Lemas, Eric L Patterson, Luan Cutti, Sarah Morran, Nicholas A Johnson, Jacob Montgomery, Fatemeh Abdollahi, David R Nelson, Victor Llaca, Kevin Fengler, Philip Westra, Todd A Gaines

**Affiliations:** Department of Agricultural Biology, Colorado State University, Fort Collins, CO 80523, USA; Department of Plant, Soil, and Microbial Science, Michigan State University, East Lansing, MI 48823, USA; Department of Plant, Soil, and Microbial Science, Michigan State University, East Lansing, MI 48823, USA; Department of Plant, Soil, and Microbial Science, Michigan State University, East Lansing, MI 48823, USA; Department of Agricultural Biology, Colorado State University, Fort Collins, CO 80523, USA; Department of Plant, Soil, and Microbial Science, Michigan State University, East Lansing, MI 48823, USA; Department of Agricultural Biology, Colorado State University, Fort Collins, CO 80523, USA; Department of Plant, Soil, and Microbial Science, Michigan State University, East Lansing, MI 48823, USA; Department of Agricultural Biology, Colorado State University, Fort Collins, CO 80523, USA; Department of Microbiology, Immunology, and Biochemistry, University of Tennessee Health Science Center, Memphis, TN 38163, USA; Corteva Agriscience, Genomics Lab, Johnston, IA 50131, USA; Corteva Agriscience, Genomics Lab, Johnston, IA 50131, USA; Department of Agricultural Biology, Colorado State University, Fort Collins, CO 80523, USA; Department of Agricultural Biology, Colorado State University, Fort Collins, CO 80523, USA

**Keywords:** Bionano, PacBio HiFi, Iso-Seq, genome assembly, Cytochrome P450, Russian thistle

## Abstract

This report presents two phased chromosome-scale genome assemblies of allotetraploid *Salsola tragus* (2n = 4x = 36) and fills the current genomics resource gap for this species. Flow cytometry estimated 1C genome size was 1.319 Gb. PacBio HiFi reads were assembled and scaffolded with Hi-C chromatin contact mapping and Bionano optical mapping data. For annotation, a PacBio Iso-Seq library was generated from root, stem, leaf, and floral tissues followed by annotation using a modified Maker pipeline. The assembled haploid *S. tragus* genomes contained 18 chromosomes each, with 9 chromosomes assigned to subgenome A and 9 chromosomes to subgenome B. Each haplome assembly represented 95% of the total flow cytometry estimated genome size. Haplome 1 and haplome 2 contained 43,354 and 42,221 annotated genes, respectively. The availability of high-quality reference genomes for this economically important weed will facilitate future omics analysis of *S. tragus* and a better understanding of chenopod plants.

**Significance**

*Salsola tragus* is a drought- and heat-tolerant invasive halophyte with high fecundity and a tumbleweed habit. Currently, few genomic resources are available to identify specific mechanisms behind this species’ resilience and adaptability. This research presents a chromosome-level reference genome for this allotetraploid species.

## Introduction

The *Salsola* genus is a member of the Chenopodioideae (goosefoot) subfamily, which is a subfamily of the broader Amaranthaceae family including beets, spinach, and quinoa, as well as the closely related weed *Bassia scoparia* (kochia). It is also distantly related to the globally important weeds *Amaranthus palmeri* (Palmer amaranth) and *Amaranthus tuberculatus* (waterhemp) (Brignone and Denham 2021). *Salsola tragus* was introduced into the United States as a contaminant in Russian imported flax seed in the late 1800s (Beckie and Francis 2009). The *Salsola* genus contains species at differing ploidy levels (2n = 18, 2n = 36, 2n = 56) (Ryan and Ayres 2000) that are highly polymorphic. Two morphologically cryptic species have been described as *S. tragus*, denoted type A and type B (Ryan and Ayres 2000). Type A was *S. tragus* first described in Eurasia with 2n = 36 chromosomes while type B is a diploid with 2n = 18 chromosomes that has not been found outside of the United States (Ryan and Ayres 2000; Sobhian et al. 2003; Smith 2005). These two cytotypes were determined to be *S. tragus* (type A) and *Salsola australis* (type B), and a third cytotype was discovered to be present throughout California and has been identified as *Salsola ryanii* (type C). *Salsola ryanii* is an allohexaploid that may have resulted from multiple separate hybridization events between *S. tragus* and *S. australis*; however, the origin of *S. australis* has yet to be determined (Hrusa and Gaskin 2008; Welles and Ellstrand 2016a, 2016b).

Chemical control of *S. tragus* in cropping systems has been effective to date; however, herbicide resistance has been reported for both of the most common herbicide modes of action, acetolactate synthase (ALS) inhibitors and glyphosate (Warwick et al. 2010; Kumar et al. 2017). Glyphosate resistance in *S. tragus* is attributed to 5-enolpyruvylshikimate-3-phosphate synthase (*EPSPS*) gene duplication in a population identified in Argentina (Yanniccari et al. 2023). Spring et al. (2022) discovered high genetic diversity within population clusters and concluded that this genetic variability can lead to an increase in the adaptability of the species. To help identify genetic mechanisms causing abiotic stress tolerance and herbicide resistance, we assembled the genome of a single *S. tragus* individual. A genetic understanding of these adaptive traits may also assist in the identification of effective control options, molecular markers for diagnostics and population monitoring, and sources of genetic variation that may improve the resilience of crops in changing climates. Our report is part of a larger effort to provide resources for the study of herbicide resistance and weed biology (Montgomery et al. 2024).

## Results and Discussion

### Genome Assembly and Annotation

We assembled two haplotypes of *S. tragus*. First, we obtained 121.7 Gb of PacBio HiFi data that was assembled and aligned to 16.15 Gb of Bionano optical map data to generate 199 hybrid scaffold maps that spanned ∼1.5 Gb. These maps were further scaffolded into pseudomolecules using 97.9 Gb of Hi-C data. One assembly was prepared for each haplotype present in somatic cells, termed haplome 1 and haplome 2. The largest pseudomolecule for each chromosome pair in terms of length was assigned to haplome 1 with the smaller being assigned to haplome 2; thus, haplome 1 may serve as a more representative sequence. Haplome 1 and Haplome 2 each consist of 18 chromosome pseudomolecules assembled from 27 and 24 phased PacBio/Bionano hybrid scaffolds, respectively, further scaffolded by Hi-C data. To facilitate the scaffolding of the allotetraploid *S. tragus* genome, a novel method for generating haplotype-specific Bionano maps was used, which when coupled with haplotype resolved contigs enabled the scaffolding and pseudomolecule creation of both subgenomes within each haplotype. The two sequence assembly lengths (Table 1) indicate that 95% of the genome was successfully assembled when compared to the flow cytometry estimated haploid genome size of 1.319 Gb per 1C. The total chromosome pseudomolecule length in haplome 1 is 1,259,258,089 bp, including 3.14 million (0.25%) ambiguous bases (N) in 221 sized gaps. The total chromosome pseudomolecule length for haplome 2 chromosome pseudomolecules is 1,247,368,765 bp, including 25.15 million (2.01%) Ns in 291 sized gaps.

**Table 1:** *Salsola tragus* genome assembly statistics and BUSCO analysis by haplome

Assembly statistics	Haplome 1	Haplome 2
Total assembly length	1,259,258,089	1,247,368,765
Scaffolds	18	18
Scaffold N50	72,893,522	72,603,936
Contig N50	11,492,176	9,056,315
Gaps	221	291
Total Ns (% of total bases)	0.25%	2.01%
Number of annotated genes	43,354	42,221
Number of telomeric ends	31 (86%)	25 (69%)
BUSCO results		
Genome (complete BUSCOs)^a^	1,571 (97.4%)	1,570 (97.2%)
Transcriptome (complete BUSCOs)^a^	1,431 (88.7%)	1,433 (88.8%)

^a^BUSCO scores generated with the embryrophyta_odb10 gene set.

Unassigned scaffolds and contigs spanning an additional 4.06 and 3.2 Mb of sequence were concatenated as chromosome 00 for haplomes 1 and 2, respectively. Chromosomes were numbered in descending size order with chromosome 1 being the largest in each assembly and chromosome 18 being the smallest. Putative centromeres were identified with CentroMiner (Lin et al. 2023). Many chromosomes had clear centromeric regions characterized by tandem repeats flanked by high repetitive element content along with reduced gene density (supplementary fig. S1, Supplementary Material online). Additional assembly statistics for both haplomes generated via Assemblathon are in Table 1 and supplementary table S1, Supplementary Material online. A python script was used to supplement Assemblathon to calculate gap number and length (Table 1; supplementary table S1, Supplementary Material online). Aside from these caveats, both haplomes contained over 95% of complete Benchmarking Universal Single-Copy Orthologs (BUSCO) genes (Table 1; supplementary table S1, Supplementary Material online). Gene models were annotated in each haplotype assembly using 4.7 Gb of PacBio Iso-Seq data along with the protein sequences from *Chenopodium quinoa* as evidence. The annotation files for haplome 1 and haplome 2 contain 43,354 and 42,221 annotated genes, respectively. BUSCO results on protein sequences generated with haplome annotation files resulted in over 88% complete BUSCOs (supplementary table S1, Supplementary Material online). Supplementary table S1, Supplementary Material online, includes both full genome and subgenome-specific BUSCO results, showing that subgenome level duplication is less than 2%, while duplication when including both subgenomes is 99.1%, indicating the completeness of gene annotation across the homoeologous chromosomes of the two subgenomes. Supplementary table S1, Supplementary Material online, also presents annotation completeness results for respective transcriptomes with just under 90% of complete BUSCOs. Haplome 1 contained 28.49% retroelements, including 21.95% Gypsy/DIRS1 LTR elements, and 3.91% DNA transposons (supplementary table S2, Supplementary Material online).

### Subgenome Assignment and Synteny Analysis

**Fig. 1.**
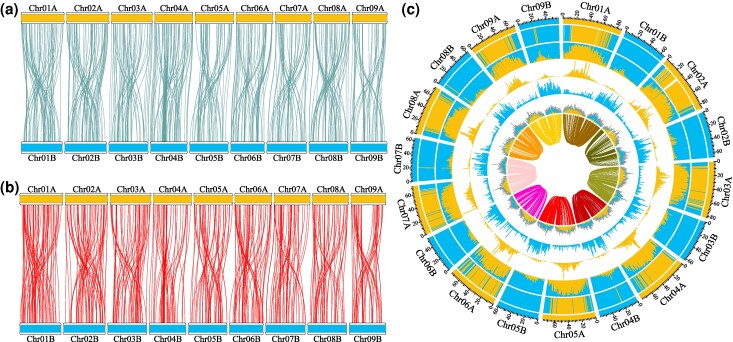
Synteny mapping between the A and B subgenomes of *S. tragus* haplome 1 with syntenic regions shown as lines (*a* = 0.7) a, b). Each line represents >95% synteny in regions greater than 1 kb. The lines in a) indicate unidirectional regions of synteny, and the lines in b) indicate an inverted region of synteny. The Circos plot from SubPhaser displays quality of chromosomal designations to each subgenome in haplome 1 c). SubPhaser designates colors to subgenomes based on a *k-*means algorithm as indicated in the outermost ring. The second level of the plot indicates significant enrichment of subgenome-specific *k*-mers. The third layer indicates relative normalized proportions of these color-coded *k*-mers. Layers 4 and 5 represent absolute counts of each *k*-mer set. Finally, the sixth layer indicates long terminal repeat retrotransposon (LTR-RT) density, where each assigned color is representative of that subgenome and gray indicates nonsignificance. Statistical significance for each of these layers is identified in 1 Mb sliding windows. The lines in the center between proposed homologs indicate syntenic regions between the two subgenomes.

SubPhaser (Jia et al. 2022) was used to identify subgenomes A and B for each haplome using kmer enrichment analysis (Fig. 1). In general, homoeologs remain intact, as shown by the lack of mixing of colors on chromosomes in Fig. 1c. However, the distal ends of several chromosomes in subgenome A contain *k*-mers that are enriched in the B subgenome, suggesting transfer of genetic material across subgenomes. Interestingly, this transfer seems to be mostly unidirectional from subgenome B to subgenome A.

### Cytochrome P450 Family Identification

Cytochrome P450s (*CYP*) have been repeatedly shown to be a key gene family for understanding broad-spectrum herbicide resistance (Gaines et al. 2020). Therefore, their identification and characterization are important for new herbicide design and discovery. As an indication of assembly and gene annotation quality, a total of 452 *CYP* candidates were identified in haplome 1 of *S. tragus* (supplementary figs. S2 and S3, Supplementary Material online). Of these, 328 were found to be full-length *CYP* genes (see supplementary fig. S2b, Supplementary Material online), with proteins ranging from 350 to 615 amino acids, while 124 were classified as fragments, having fewer than 350 amino acids (supplementary tables S3 and S4, Supplementary Material online). All 328 *S. tragus CYP* gene IDs for haplome 1 are listed in supplementary table S4, Supplementary Material online.

## Materials and Methods

### Sample Preparation, DNA Extraction, and Sequencing

All materials for sequencing were gathered from one individual obtained from an herbicide susceptible *S. tragus* population collected in Weld County, Colorado. Fresh tissue was used for Bionano optical mapping, and flash-frozen young tissue was used for PacBio HiFi sequencing and Hi-C chromatin contact mapping. Flash-frozen tissues of roots, stems, young leaves, and flowers were used for RNA extraction and PacBio Iso-Seq. Flash-frozen tissue and RNA samples were shipped on dry ice, and Bionano tissue samples were shipped at 4 °C to the Genome Center of Excellence at Corteva Agriscience for high molecular weight DNA extraction, library preparation, and sequencing as detailed in the Supplementary material.

### Flow Cytometry Estimated Genome Size

The estimated genome size was evaluated at the Flow Cytometry Facility of the Iowa State University Office of Biotechnology. Using *Zea mays* as an internal standard, the average genome size for four biological replicates was 1.319 Gb per 1C. Chromosome counts through hybridization and imaging demonstrated the genome of this *S. tragus* individual contains 2n = 4x = 36 chromosomes per somatic cell (Montgomery et al. 2024).

### Genome Assembly and Annotation

Genome assembly was performed as described in the Supplementary material using phased hybrid scaffolding with a combination of Bionano optical genome mapping, PacBio HiFi long-read sequencing, and Hi-C Seq chromatin conformation. PacBio HiFi reads were assembled using Hifiasm (Cheng et al. 2021) and aligned to the Bionano map set, producing hybrid scaffolds that were validated as pseudomolecules using the Hi-C data. As *S. tragus* is an outcrossing polyploid, modifications were made to the Bionano “haplotype-aware” genome maps that have been optimized for the human genome resulting in a novel assembly approach that overcomes common obstacles in this system. This novel method is based on the alignment of Direct Label Enzyme (DLE-1) labeled molecules to a combined assembly containing both Hi-C phased haplotypes from a PacBio HiFi Hifiasm (Cheng et al. 2021) contig assembly (in the case of polyploids, each “haplotype” may contain multiple subgenomes). From this alignment, haplotype-specific molecules can be obtained and used to generate haplotype-specific genome maps, which in turn can be used to generate hybrid scaffolds with a haplotype match pair of contigs and maps (detailed description in supplementary methods S1, Supplementary Material online).

Gene annotation using PacBio Iso-Seq transcript data along with UniProt and Arabidopsis databases was conducted as described in the Supplementary material. Briefly, repeats were identified with RepeatModeler (v.2.0.2) (Flynn et al. 2020) and softmasked with RepeatMasker (v.4.1.2) (http://www.repeatmasker.org/RepeatMasker/). Iso-Seq reads from root, leaf, stem, and floral tissue were aligned to the softmasked assemblies with Minimap2 (Li 2018). MakerP (v.1.0) (Cantarel et al. 2008) was used to combine evidence from Iso-Seq alignments and protein sequences from *C. quinoa* (Jarvis et al. 2017) to produce a final gene set for each haplome. Gene models were assigned functional information through alignment to several databases including InterPro, UniRef, and the NCBI nonredundant protein databases. Both assemblies were analyzed using the BUSCO (v.4.0.2) embryophyta_odb10 gene set, which included 31 species and 1,614 BUSCOs (Manni et al. 2021). Protein files for further BUSCO analyses of annotated transcripts were generated using GffRead (v.0.12.7) (Pertea and Pertea 2020). Cytochrome P450 classification methods are detailed in supplementary methods S1, Supplementary Material online. Karyotype and annotation feature files were generated for use in RStudio RIdeogram (RStudio v4.2.1, RIdeogram v0.2.2) for genome visualization (Hao et al. 2020). EDTA (v.2.2.0) (Su et al. 2021) and SubPhaser (v1.2.6) (Jia et al. 2022) were used to identify repetitive elements (supplementary table S2, Supplementary Material online) and visualize assembly quality. Finally, assembly statistics were gathered through Assemblathon (Perl v5.34.1) (Earl et al. 2011).

### Subgenome Phasing and Synteny Analysis

Karyotype and annotation feature files were generated for use in RIdeogram (v.0.2.2) for genome visualization (Hao et al. 2020). SubPhaser (v1.2.6) (Jia et al. 2022) was used to identify *k*-mers enriched in each subgenome and to separate homoeologous chromosomes into their respective subgenomes. Haplome 1 was indexed using SAMtools (v1.16.1) (Li et al. 2009) and split into A and B chromosomes. Indexed B chromosomes were then used in Minimap2 (v2.24) (Li 2018) to align to the A chromosomes. The resulting .paf file was converted to a .coords file through ragtag (v2.1.0) (Alonge et al. 2022) and show-coords (mummer, v3.23) (Marçais et al. 2018). The output file was used to identify syntenic regions between the A and B chromosomes (≥1 kb, ≥95% synteny). These syntenic regions were visualized using RIdeogram (v.0.2.2) (Hao et al. 2020).

## Supplementary Material

evaf014_Supplementary_Data

## Data Availability

Assemblies and annotations for both the reference (haplome 1) and alternative (haplome 2) haplomes are available on Weedpedia (https://weedpedia.weedgenomics.org/) and NCBI under BioProject PRJNA1153015 with genome accession numbers JBJQNN000000000 and JBJQNO000000000. Sequencing reads and other data used in the assembly and annotation are available through NCBI under the umbrella project PRJNA1159769 and SRA accessions SRR30621585, SRR30621586, and SRR30621587.
