# Supplementary material for: Assembly and Annotation of the Tetraploid Salsola tragus (Russian Thistle) Genome
Source: Genome Biol Evol. 2025 Jan 27;17(2):evaf014. doi: 10.1093/gbe/evaf014 (PMC11797066; doi:10.1093/gbe/evaf014)
Supplement: evaf014_Supplementary_Data [file evaf014_supplementary_data.docx]

**Supplemental Information**

**Methods**

**Sample Preparation, DNA Extraction, and Sequencing**

Seeds from a *Salsola tragus* accession collected in Weld County, Colorado, USA were sown in presoaked potting soil (Lambert LM-GPS) and watered through subirrigation. Upon reaching ~5 cm in height, seedlings were transplanted to 16-cm pots (America Clay Works I-A650MP) filled with the same soil and grown under greenhouse conditions (25/20 ºC and 16/8-h day/night cycles). A single flowering plant was placed in the dark for 72 h, after which 4 g of fresh leaf tissue was sampled for PacBio HiFi library preparation and 2 g of fresh leaf tissue for Hi-C library preparation. In each case, tissue was flash frozen in liquid nitrogen and stored at -80 ºC until use. The same individual plant was used to harvest 4 g of fresh tissue that was immediately shipped on damp paper towels at 4 ºC for Bionano library preparation. Six additional plants were grown as previously described. Root, stem, leaf, and meristem tissue was sampled from young (~6-10 leaf) and old (flowering) plants and combined with floral tissue from all stages of development for RNA extraction (Zymo Direct-zol RNA Miniprep). Following quantification of concentration and quality, 3 µg of RNA was used for PacBio Iso-Seq library preparation. All samples were shipped on dry ice (except for Bionano tissue, which was shipped at 4 ºC) to the Genome Center of Excellence at Corteva Agriscience for DNA extraction, library preparation, and sequencing.

The *Salsola tragus* genome was constructed using a combination of Bionano Optical genome mapping and HiFi PacBio long sequencing as well as Hi-C Seq Illumina short read data. Bionano mapping was performed as previously described (Hufford et al. 2021), with some modifications. Approximately 500 mg of young leaf tissue were fixed in 2% formaldehyde, chopped, homogenized, and filtered through 40 and 100 µm cell strainers, according to manufacturer’s protocol using the Prep™ Plant Tissue DNA Isolation Kit (Bionano Genomics, San Diego CA). The resulting nuclei were resuspended and cleaned from debris and other solids using a series of low-speed centrifugations (100 x g) followed by centrifugation at 2,500 x g to concentrate and wash nuclei. Nuclei were resuspended, centrifugated again at 2,500 x g, and embedded in low melting point agarose. The resulting ultra-high molecular weight (uHMW) DNA was recovered from the agarose plug by melting it at 65ºC, followed by incubation in agarose at 43 ºC and drop dialysis against TE buffer.

For PacBio HiFi sequencing, DNA was isolated from approximately 1 g frozen leaf tissue using the Nucleobond HMW DNA kit (Macherey-Nagel) following the manufacturer’s protocol. Purified DNA was sheared to a narrow size distribution of 10 to 20 kb with a Megaruptor 3 system (Diagenode) set at speed 34. The sheared DNA was used to construct a SMRTBell HiFi library using the Express Template Prep Kit 2.0 (PN: 101-853-100) as per manufacturers protocol. DNA fragments were size selected using the 15-20 kb High Pass 75E protocol in a PippinHT System (Sage Science; PN: HPE-7510). The resulting SMRTbell library was bound to the sequencing polymerase enzyme and ran in two SMRTCells in a PacBio Sequel IIe sequencer, yielding a total of 121.7 Gbp of HiFi reads.

One Hi-C Seq chromatin conformation capture library was constructed using the Phase Genomics Proximo system, as per manufacturer’s protocol (Cat. KT3040; Phase Genomics, Seattle, WA). The library was constructed from chromatin crosslinked and isolated from 1 g of frozen leaf tissue as per manufacturer’s protocol. The amplified library was sequenced in an Illumina NovaSeq 6000 system yielding a total sequence length of 97.9 Gbp.

**Creation of Haplotype-Specific Bionano Genome Maps**

The Bionano Direct Label and Stain (DLS) protocol was performed using an 800-ng uHMW with a DLS Kit, as per manufacturer’s protocol (PN 80005), with some modifications. DNA was incubated in the presence of DLE-1 Enzyme, DL-Green and DLE-1 Buffer for 3 hours 40 minutes at 37 ºC and later incubated with Proteinase K for 1 hour at 50 ºC. After dye cleaning the reaction was stained, diluted in flow buffer, and loaded into a single Bionano chip in a Saphyr system as per manufacturers recommendations. Genome map assembly and hybrid scaffold construction were performed using Bionano Access version 1.7 and Tools 3.7 in a local Bionano Computer server. To facilitate the scaffolding of the allotetraploid *Salsola tragus* genome, a novel method for generating haplotype-specific Bionano maps was used, which when coupled with haplotype resolved contigs enabled the scaffolding and pseudomolecule creation of all four haplotypes present in the sample. The Bionano ecosystem has a concept of ‘haplotype-aware’ genome maps that has been optimized for human genome structural variation analysis relative to reference genome (Bionano Solve Theory of Operation: Structural Variant Calling). However, this concept does not apply to the hybrid scaffolding of either primary/alternative or haplotype-resolved assemblies as the Bionano genome maps may contain irreconcilable phase switches in regions of the genome that contain identical or similar Direct Label Enzyme (DLE-1) labeling patterns. To overcome this obstacle, a novel method of creating haplotype-specific maps was developed. This method is based on the alignment of DLE-1 labeled molecules to a combined assembly containing both Hi-C phased haplotypes from a PacBio HiFi hifiasm contig assembly (in the case of polyploids, each “haplotype” group may contain multiple subgenomes). From this alignment, haplotype-specific molecules can be obtained and used to generate haplotype-specific genome maps, which in turn can be used to generate hybrid scaffolds with a haplotype match pair of contigs and maps. This approach relies on two key components, 1) molecules preferentially align to the best haplotype contig match and 2) molecules with overhanging alignments are captured, which will enable the downstream creation of contig spanning maps. Most of the steps of this method can be done within the Bionano Access platform, but one step is done from the command-line using Bionano Solve tools. The specific details for these methods were designed by and proprietary to Corteva Agriscience. The preliminary, non-phased assembly of the filtered molecules resulted in 199 genome optical maps spanning roughly 1.5 Gbp.

**Genome Annotation**

The two chromosome-level haplotype assemblies had the chromosomes sorted by size and were annotated individually. *De novo* transposable element families in the two assemblies were identified using RepeatModeler (v.2.0.2) (Flynn et al. 2020), and the assemblies were softmasked using RepeatMasker (v.4.1.2) (<http://www.repeatmasker.org/RepeatMasker/>) to generate an annotation of repeat elements and BEDtools (v.2.30.0) (Quinlan & Hall) to softmask the genome using that annotation. The Iso-Seq reads were mapped to the softmasked genome using pbmm2 (v.1.10.0). Redundant transcripts were collapsed to unique isoforms using IsoSeq3 (v.3.8.2) (<https://github.com/PacificBiosciences/IsoSeq>). The final gene model predictions were made using MakerP (v.1.0) (Cantarel et al. 2008), utilizing the collapsed gene predictions from IsoSeq3 and all annotated protein sequences from  *Chenopodium quinoa* (Jarvis et al. 2017) available on NCBI. Predicted small proteins (<12 amino acids) were filtered out and the remaining gene models were renumbered using a standard nomenclature. Functional annotation of the gene models was prescribed using a series of tools that predict protein location, function, and homology. MultiLoc2 (v.1.0) (Blum et al. 2009) was used to predict the subcellular localization of all proteins and whether or not they had a secretory sequence and/or transmembrane domains. InterProScan5 (v.5.47-82.0) (Jones et al. 2014) and Uniprot (The UniProt Consortium et al. 2023) were used to predict protein domains within all the proteins and prescribe Interpro IDs, GOterms, Pather IDs, Kegg Terms, etc. MMSeqs2 (v.4.1) (Steinegger & Söding 2017) was used to search both the Uniref50 database from UniProt and a custom NCBI protein database for the nearest annotated homologue (i.e. best hit) to prescribe function by whole-protein homology. The custom NCBI protein database contained a manually curated list of proteins that are known herbicide targets. The best hit to the Uniref50 database was converted into the KEGG Orthology (KO) ID (Kanehisa et al. 2017) and prescribed. The genome annotation (i.e. the gff) was modified to include relevant functional information in the notes column for each gene including the Interpro ID, GO IDs, and the closest known annotated protein.

**Cytochrome P450 Family Identification**

Cytochrome P450s (*CYP*) have been repeatedly shown to be a key gene family for understanding broad spectrum herbicide resistance (Gaines et al. 2020). Therefore, their identification and characterization is important for new herbicide design and discovery. A total of 452 *CYP* candidates were identified in haplome 1 of *S. tragus* (Figure S2, S3) and named following the Standardized Cytochrome Nomenclature Committee's guidelines (http://drnelson.uthsc.edu/CytochromeP450.html). These candidates were divided into 47 families and 77 subfamilies (Table S3, S4). These P450 genes were categorized into two major clades: the A type, which included the CYP71 clan, and the non-A type, comprising seven clans (Figure S2A). Of these, 328 were found to be full-length *CYP* genes (see Figure S2B), with proteins ranging from 350 to 615 amino acids, while 124 were classified as fragments, having fewer than 350 amino acids (Table S3, S4). All 328 *Salsola tragus CYP* gene IDs for haplome 1 are listed in Table S4.

The identification of cytochrome P450 genes (*CYP*) in the annotated protein sequences of *S. tragus* haplome 1 utilized the InterPro codes "IPR001128" and "IPR036396". All identified protein candidates were submitted to the Standardized Cytochrome Nomenclature Committee to maintain consistent naming conventions (<http://drnelson.uthsc.edu/CytochromeP450.html>). The chromosomal mapping of *S. tragus* *CYP* genes was based on karyotype, gene density, and physical location information from the *S. tragus* haplome 1 genome annotation file, employing the R package RIdeogram (Hao et al., 2020). The amino acid sequences of *S. tragus* *CYP* proteins were aligned using ClustalW (Thompson et al. 1994). A neighbor-joining (NJ) phylogenetic tree of 328 *S. tragus* CYP proteins was constructed using MEGAX software with 1000 bootstrap replications (Kumar et al., 2016). The resulting phylogenetic tree was visualized using the ITOL9 web server (<https://itol.embl.de/>).

**Supplementary References**

Blum T, Briesemeister S, Kohlbacher O. 2009. MultiLoc2: integrating phylogeny and Gene Ontology terms improves subcellular protein localization prediction. BMC Bioinformatics. 10:274. doi: 10.1186/1471-2105-10-274.

Cantarel BL et al. 2008. MAKER: An easy-to-use annotation pipeline designed for emerging model organism genomes. Genome Res. 18:188–196. doi: 10.1101/gr.6743907.

Flynn JM et al. 2020. RepeatModeler2 for automated genomic discovery of transposable element families. Proc. Natl. Acad. Sci. U.S.A. 117:9451–9457. doi: 10.1073/pnas.1921046117.

Gaines TA et al. 2020. Mechanisms of evolved herbicide resistance. Journal of Biological Chemistry. 295:10307–10330. doi: 10.1074/jbc.REV120.013572.

Hufford MB et al. 2021. De novo assembly, annotation, and comparative analysis of 26 diverse maize genomes. Science. 373:655-662.

Jones P et al. 2014. InterProScan 5: genome-scale protein function classification. Bioinformatics. 30:1236–1240. doi: 10.1093/bioinformatics/btu031.

Kanehisa M, Furumichi M, Tanabe M, Sato Y, Morishima K. 2017. KEGG: new perspectives on genomes, pathways, diseases and drugs. Nucleic Acids Res. 45:D353–D361. doi: 10.1093/nar/gkw1092.

Quinlan AR, Hall IM. 2010. BEDTools: a ﬂexible suite of utilities for comparing genomic features. Bioinformatics. 26:841-842.

Steinegger M, Söding J. 2017. MMseqs2 enables sensitive protein sequence searching for the analysis of massive data sets. Nat Biotechnol. 35:1026–1028. doi: 10.1038/nbt.3988.

The UniProt Consortium et al. 2023. UniProt: the Universal Protein Knowledgebase in 2023. Nucleic Acids Research. 51:D523–D531. doi: 10.1093/nar/gkac1052.

Thompson JD, Higgins DG, Gibson TJ. 1994. CLUSTAL W: improving the sensitivity of progressive multiple sequence alignment through sequence weighting, position-specific gap penalties and weight matrix choice. OUP. 22. doi: 10.1093/nar/22.22.4673.

**Figure S1:** These plots display putative centromeres for each individual chromosome as identified by CentroMiner. Many chromosomes had clear centromeric regions characterized by tandem repeats flanked by high repetitive element content along with reduced gene density.

**Figure S2:** A simplified phylogenetic tree of Cytochrome P450 (*CYP*) gene families from *S. tragus* (**A**) and the genomic locations of the 328 *CYP* genes identified in the haplome 1 assembly of *S. tragus* (**B**)*.* The number of *CYP* genes in each branch is indicated in parenthesis (**A**), and *CYP* gene density across all 18 chromosomes is also displayed as a heatmap overlayed onto the karyotype figure (**B**). Further details for these identified *CYP* families and subfamilies can be found in Figure S3 and tables S3 and S4.

**Figure S3:** This figure displays the eight clan designations for all 328 full length cytochrome P450 (*CYP*) genes in the haplome 1 reference genome and provides further detail on the relatedness of each of the *CYP* genes that was not included in Figure S2A.

**Table S1.** *Salsola tragus* genome assembly statistics and BUSCO analysis by haplome.

| Assembly | | Haplotype 1 | | | | Haplotype 2 | | | | | | | | | | | | |  |
| --- | --- | --- | --- | --- | --- | --- | --- | --- | --- | --- | --- | --- | --- | --- | --- | --- | --- | --- | --- |
| Number of scaffolds | | 19 | | | | 19 | | | | | | | | | | | | |  |
| Total size of scaffolds | | 1,263,315,787 | | | | 1,250,604,984 | | | | | | | | | | | | |  |
| Longest scaffold | | 86,803,187 | | | | 86,596,344 | | | | | | | | | | | | |  |
| Shortest scaffold | | 4,057,698 | | | | 3,236,219 | | | | | | | | | | | | |  |
| Number of gaps | | 255 | | | | 319 | | | | | | | | | | | | |  |
| Average gap size | | 12,348 | | | | 78,859 | | | | | | | | | | | | |  |
| Gap N50 | | 73,219 | | | | 351,468 | | | | | | | | | | | | |  |
| Number of scaffolds > 10M nt | | 18 | | | | 18 | | | | | | | | | | | | |  |
| Percentage of scaffolds > 10M nt | | 94.7 | | | | 94.7 | | | | | | | | | | | | |  |
| Mean scaffold size | | 66,490,305 | | | | 65,821,315 | | | | | | | | | | | | |  |
| Median scaffold size | | 67,360,422 | | | | 66,189,443 | | | | | | | | | | | | |  |
| N50 scaffold length | | 70,363,782 | | | | 72,603,936 | | | | | | | | | | | | |  |
| L50 scaffold count | | 9 | | | | 8 | | | | | | | | | | | | |  |
| scaffold %A | | 31.78 | | | | 31.23 | | | | | | | | | | | | |  |
| scaffold %C | | 18.07 | | | | 17.74 | | | | | | | | | | | | |  |
| scaffold %G | | 18.09 | | | | 17.76 | | | | | | | | | | | | |  |
| scaffold %T | | 31.82 | | | | 31.26 | | | | | | | | | | | | |  |
| scaffold %N | | 0.25 | | | | 2.01 | | | | | | | | | | | | |  |
| N50 contig length | | 11,307,909 | | | | 9,056,315 | | | | | | | | | | | | |  |
| L50 contig count | | 35 | | | | 40 | | | | | | | | | | | | |  |
| Complete BUSCO Results | | | | | | | | | | | | | | | |  |  |  |  |
|  | | Haplotype 1 | | | | Haplotype 2 | | | | | | | | | | | | |  |
|  | | Genome | | | Transcriptome | | | | | | Genome | | | | Transcriptome | | | | |
| Complete | | 1,571 (97.4%) | | | 1,431 (88.7%) | | | | | | 1,570 (97.2%) | | | | 1,433 (88.8%) | | | | |
| Complete and Single-copy | | 287 (17.8%) | | | 282 (17.5%) | | | | | | 351 (21.7%) | | | | 341 (21.1%) | | | | |
| Complete and Duplicated | | 1,284 (79.6%) | | | 1,149 (71.2%) | | | | | | 1,219 (75.5%) | | | | 1,092 (67.7%) | | | | |
| Fragmented | | 9 (0.06%) | | | 83 (5.1%) | | | | | | 10 (0.06%) | | | | 83 (5.1%) | | | | |
| Missing | | 34 (2.0%) | | | 100 (6.2%) | | | | | | 34 (2.2%) | | | | 98 (6.1%) | | | | |
| Searched | | 1,614 | | | | 1,614 | | | | | | | | | | | | |  |
| Subgenome-specific Results | | | | | | | | | | | | | | | | |  |  |  |
| Haplotype 1 | Predicted | | | | | | | | Annotated | | | | | | | |  |  |  |
|  | A Subgenome | | B Subgenome | | | | | A Subgenome | | | | | B Subgenome | | | | |  |  |
| Complete | 1,578 (97.7%) | | 1,583 (98.1%) | | | | | 1,353 (83.9%) | | | | | 1,345 (83.3%) | | | | |  |  |
| Complete - Single Copy | 1,553 (96.2%) | | 1,553 (96.2%) | | | | | 1,339 (83.0%) | | | | | 1,322 (81.9%) | | | | |  |  |
| Complete - Duplicated | 25 (1.5%) | | 30 (1.9%) | | | | | 14 (0.9%) | | | | | 23 (1.4%) | | | | |  |  |
| Fragmented | 10 (0.6%) | | 11 (0.7%) | | | | | 142 (8.8%) | | | | | 148 (9.2%) | | | | |  |  |
| Missing | 26 (1.7% | | 20 (1.2%) | | | | | 119 (7.3%) | | | | | 121 (7.5%) | | | | |  |  |
| Haplotype 2 | Predicted | | | | | | Annotated | | | | | | | | | | |  |  |
|  | A Subgenome | | | B Subgenome | | | | | | A Subgenome | | | | B Subgenome | | | |  |  |
| Complete | 1,558 (96.5%) | | | 1,539 (95.4%) | | | | | | 1,329 (82.3%) | | | | 1,312 (81.3%) | | | |  |  |
| Complete - Single Copy | 1,529 (94.7%) | | | 1,517 (94.0%) | | | | | | 1,311 (81.2%) | | | | 1,293 (80.1%) | | | |  |  |
| Complete - Duplicated | 29 (1.8%) | | | 22 (1.4%) | | | | | | 18 (1.1%) | | | | 19 (1.2%) | | | |  |  |
| Fragmented | 13 (0.8%) | | | 12 (0.8%) | | | | | | 144 (8.9%) | | | | 140 (8.7%) | | | |  |  |
| Missing | 43 (2.7%) | | | 63 (3.9%) | | | | | | 141 (8.8%) | | | | 162 (10.0%) | | | |  |  |
| Full Polyploid Genome Results | | | | | | | | | | | | | | | | | |  |  |
|  | | Predicted | | | | | | | | | | Annotated | | | | | | |  |
| Complete | | 1,599 (99.1%) | | | | | | | | | | 1,472 (91.2%) | | | | | | |  |
| Complete - Single Copy | | 8 (0.05%) | | | | | | | | | | 20 (1.2%) | | | | | | |  |
| Complete - Duplicated | | 1,591 (98.6%) | | | | | | | | | | 1,452 (90%) | | | | | | |  |
| Fragmented | | 4 (0.02%) | | | | | | | | | | 80 (5.0%) | | | | | | |  |
| Missing | | 11 (0.7%) | | | | | | | | | | 62 (3.8%) | | | | | | |  |

**Table S2.** *Salsola tragus* Haplome 1 Repetitive Genetic Elements as identified by EDTA.

| Interspersed Repeats |  | Count | Length (BP) | Percentage |
| --- | --- | --- | --- | --- |
| LINEs: | CRE/SLACS | 4,475 | 3,077,879 | 0.24 |
|  | L2/CR1/Rex | 22,925 | 2,539,615 | 0.2 |
|  | RTE/Bov-B | 7,589 | 3,771,845 | 0.3 |
|  | L1/CIN4 | 26,784 | 23,711,183 | 1.88 |
| LTR Elements | BEL/Pao | 190 | 239,885 | 0.02 |
|  | Ty1/Copia | 39,786 | 42,474,513 | 3.36 |
|  | Gypsy/DIRS1 | 168,484 | 277,354,277 | 21.95 |
|  | Retroviral | 1,876 | 869,046 | 0.07 |
| Total Retroelements |  | 279,510 | 359,864,068 | 28.49 |
| DNA transposons | hobo-Activator | 13,740 | 4,583,526 | 0.36 |
|  | Tc1-IS630-Pogo | 6,759 | 2,157,734 | 0.17 |
|  | Tourist/Harbinger | 9,366 | 4,866,971 | 0.39 |
| Total Transposons |  | 90,299 | 49,450,348 | 3.91 |
| Other | Rolling-circles | 3,538 | 2,673,826 | 0.21 |
|  | Unclassified: | 1,108,117 | 347,287,657 | 27.49 |
| Total Interspersed Repeats |  |  | 756,602,073 | 59.89 |

**Table S3**. Information on *Salsola tragus* Cytochrome P450 genes in haplome 1.

| P450 Family | No. of P450 per families | No. of P450 subfamilies | P450 subfamilies | No. of P450 per subfamilies | P450 fragments per subfamilies |
| --- | --- | --- | --- | --- | --- |
| CYP71 | 104 | 6 | AH | 1 | - |
|  |  |  | AT | 30 | 7 |
|  |  |  | AP | 2 | - |
|  |  |  | AU | 28 | 4 |
|  |  |  | BE | 29 | 9 |
|  |  |  | FC | 13 | 2 |
|  |  |  | - | 1 | 1 |
| CYP72 | 42 | 3 | A | 38 | 9 |
|  |  |  | D | 2 | - |
|  |  |  | Q | 2 | 1 |
| CYP73 | 2 | 1 | A | 2 | - |
| CYP74 | 7 | 2 | A | 5 | 1 |
|  |  |  | B | 2 | 1 |
| CYP75 | 5 | 1 | B | 5 | 1 |
| CYP76 | 39 | 4 | A | 2 | - |
|  |  |  | AD | 29 | 7 |
|  |  |  | F | 6 | 3 |
|  |  |  | BN | 2 | 1 |
| CYP77 | 5 | 2 | A | 2 | 1 |
|  |  |  | B | 3 | 1 |
| CYP78 | 9 | 1 | A | 9 | 1 |
| CYP81 | 32 | 9 | B | 4 | - |
|  |  |  | BG | 6 | 2 |
|  |  |  | CJ | 6 | 5 |
|  |  |  | CL | 3 | 2 |
|  |  |  | CR | 2 | - |
|  |  |  | CS | 3 | 1 |
|  |  |  | CT | 4 | 1 |
|  |  |  | DF | 2 | - |
|  |  |  | DG | 2 | - |
| CYP82 | 33 | 3 | D | 26 | 12 |
|  |  |  | U | 2 | - |
|  |  |  | BJ | 5 | 3 |
| CYP85 | 1 | 1 | A | 1 | - |
| CYP86 | 12 | 3 | A | 4 | 2 |
|  |  |  | B | 4 | - |
|  |  |  | C | 4 | - |
| CYP87 | 4 | 2 | A | 2 | - |
|  |  |  | D | 2 | - |
| CYP88 |  | 1 | A | 7 | - |
| CYP89 | 10 | 1 | A | 7 | 7 |
|  |  |  | - | 1 | 1 |
| CYP90 | 8 | 4 | A | 2 | - |
|  |  |  | B | 2 | - |
|  |  |  | C | 2 | - |
|  |  |  | D | 2 | - |
| CYP92 | 3 | 1 | A | 3 | - |
| CYP94 | 15 | 4 | A | 5 | 3 |
|  |  |  | B | 2 | - |
|  |  |  | C | 4 | - |
|  |  |  | D | 4 | 2 |
| CYP96 | 30 | 3 | A | 27 | 13 |
|  |  |  | AJ | 2 | 3 |
|  |  |  | AK | 1 | 1 |
| CYP97 | 6 | 3 | A | 2 | - |
|  |  |  | B | 2 | - |
|  |  |  | C | 2 | - |
| CYP98 | 15 | 1 | A | 15 | 7 |
| CYP701 | 2 | 1 | A | 2 | - |
| CYP703 | 1 | 1 | A | 1 |  |
| CYP704 | 8 | 2 | A | 6 | 2 |
|  |  |  | B | 2 | - |
| CYP706 | 5 | 2 | C | 2 | - |
|  |  |  | Z | 3 | - |
| CYP707 | 6 | 1 | A | 6 | 2 |
| CYP710 | 2 | 1 | A | 2 | - |
| CYP711 | 1 | 1 | A | 1 | - |
| CYP712 | 2 | 1 | D | 2 | - |
| CYP714 | 5 | 2 | A | 2 | - |
|  |  |  | E | 3 | 2 |
| CYP715 | 2 | 1 | A | 2 | - |
| CYP716 | 12 | 2 | A | 9 | 1 |
|  |  |  | CL | 3 | 1 |
| CYP718 | 2 | 1 | A | 2 | - |
| CYP721 | 2 | 1 | A | 2 | - |
| CYP722 | 2 | 1 | A | 2 | - |
| CYP734 | 3 | 1 | A | 3 | - |
| CYP735 | 4 | 1 | A | 4 | - |
| CYP736 | 4 | 1 | T | 4 | 1 |
| TOTAL | 452 | 77 | - | 452 | 124 |

**Table S4.** *Salsola tragus* cytochrome P450 gene IDs in haplome 1 and their names as reported in Figure S3.

| *Salsola tragus* P450 gene IDs | | |
| --- | --- | --- |
| 1 | CYP71AH56_8A | SalTrChr08Ag193000 |
| 2 | CYP71AP62_8B | SalTrChr08Bg408330 |
| 3 | CYP71AP62_8A | SalTrChr08Ag192940 |
| 4 | CYP71AT294_3B | SalTrChr03Bg274090 |
| 5 | CYP71AT295_8A | SalTrChr08Ag173600 |
| 6 | CYP71AT295_8B | SalTrChr08Bg388650 |
| 7 | CYP71AT296_8A | SalTrChr08Ag173610 |
| 8 | CYP71AT297_8A | SalTrChr08Ag173620 |
| 9 | CYP71AT298_8B | SalTrChr08Bg388670 |
| 10 | CYP71AT300_9A | SalTrChr09Ag208440 |
| 11 | CYP71AT300_9B | SalTrChr09Bg423250 |
| 12 | CYP71AT301_9A | SalTrChr09Ag208460 |
| 13 | CYP71AT301_9B | SalTrChr09Bg423270 |
| 14 | CYP71AT302a_9A | SalTrChr09Ag208470 |
| 15 | CYP71AT302b_9A | SalTrChr09Ag208530 |
| 16 | CYP71AT302_9B | SalTrChr09Bg423280 |
| 17 | CYP71AT304_9A | SalTrChr09Ag208540 |
| 18 | CYP71AT304_9B | SalTrChr09Bg423290 |
| 19 | CYP71AT305_9A | SalTrChr09Ag208550 |
| 20 | CYP71AT306_9A | SalTrChr09Ag208560 |
| 21 | CYP71AT307_9A | SalTrChr09Ag208590 |
| 22 | CYP71AT308_9A | SalTrChr09Ag208610 |
| 23 | CYP71AT309_9A | SalTrChr09Ag208620 |
| 24 | CYP71AT309_9B | SalTrChr09Bg423330 |
| 25 | CYP71AT310_9A | SalTrChr09Ag208640 |
| 26 | CYP71AT311_9B | SalTrChr09Bg423300 |
| 27 | CYP71AU245_3B | SalTrChr03Bg281650 |
| 28 | CYP71AU247_8A | SalTrChr08Ag190920 |
| 29 | CYP71AU247_8B | SalTrChr08Bg397420 |
| 30 | CYP71AU248_8A | SalTrChr08Ag190930 |
| 31 | CYP71AU248_8B | SalTrChr08Bg397410 |
| 32 | CYP71AU429_8A | SalTrChr08Ag190940 |
| 33 | CYP71AU429_8B | SalTrChr08Bg397400 |
| 34 | CYP71AU430_8A | SalTrChr08Ag192840 |
| 35 | CYP71AU430_8B | SalTrChr08Bg408240 |
| 36 | CYP71AU431_8A | SalTrChr08Ag192850 |
| 37 | CYP71AU431_8B | SalTrChr08Bg408250 |
| 38 | CYP71AU432_8A | SalTrChr08Ag192860 |
| 39 | CYP71AU433_8A | SalTrChr08Ag192890 |
| 40 | CYP71AU434_8A | SalTrChr08Ag192900 |
| 41 | CYP71AU435_8A | SalTrChr08Ag192910 |
| 42 | CYP71AU435_8B | SalTrChr08Bg408360 |
| 43 | CYP71AU436_8A | SalTrChr08Ag192920 |
| 44 | CYP71AU436_8B | SalTrChr08Bg408370 |
| 45 | CYP71AU437_8A | SalTrChr08Ag192930 |
| 46 | CYP71AU437_8B | SalTrChr08Bg408320 |
| 47 | CYP71AU438_8B | SalTrChr08Bg397430 |
| 48 | CYP71AU439_8B | SalTrChr08Bg408220 |
| 49 | CYP71AU440_8B | SalTrChr08Bg408230 |
| 50 | CYP71AU441_9A | SalTrChr09Ag193520 |
| 51 | CYP71BE225_1A | SalTrChr01Ag002030 |
| 52 | CYP71BE225_5B | SalTrChr05Bg330940 |
| 53 | CYP71BE226_1A | SalTrChr01Ag005940 |
| 54 | CYP71BE226_1B | SalTrChr01Bg222810 |
| 55 | CYP71BE227_1A | SalTrChr01Ag016040 |
| 56 | CYP71BE227_1B | SalTrChr01Bg233210 |
| 57 | CYP71BE228_1A | SalTrChr01Ag016070 |
| 58 | CYP71BE229_1A | SalTrChr01Ag016080 |
| 59 | CYP71BE229_1B | SalTrChr01Bg233300 |
| 60 | CYP71BE230_1A | SalTrChr01Ag016090 |
| 61 | CYP71BE230_1B | SalTrChr01Bg233270 |
| 62 | CYP71BE231_1B | SalTrChr01Bg233260 |
| 63 | CYP71BE232_1B | SalTrChr01Bg233280 |
| 64 | CYP71BE233_1B | SalTrChr01Bg233310 |
| 65 | CYP71BE234_2B | SalTrChr02Bg263530 |
| 66 | CYP71BE235_6B | SalTrChr06Bg352970 |
| 67 | CYP71BE236_8B | SalTrChr08Bg389800 |
| 68 | CYP71BE237_8A | SalTrChr08Ag174660 |
| 69 | CYP71BE237_8B | SalTrChr08Bg389770 |
| 70 | CYP71BE238_8B | SalTrChr08Bg389760 |
| 71 | CYP71FC5_2A | SalTrChr02Ag046840 |
| 72 | CYP71FC5_2B | SalTrChr02Bg263630 |
| 73 | CYP71FC6_2A | SalTrChr02Ag046850 |
| 74 | CYP71FC6_2B | SalTrChr02Bg263660 |
| 75 | CYP71FC7_2A | SalTrChr02Ag046950 |
| 76 | CYP71FC7_2B | SalTrChr02Bg263590 |
| 77 | CYP71FC8_2B | SalTrChr02Bg263600 |
| 78 | CYP71FC9_2B | SalTrChr02Bg263610 |
| 79 | CYP71FC10_2B | SalTrChr02Bg263640 |
| 80 | CYP71FC11_7A | SalTrChr07Ag147800 |
| 81 | CYP71FC12_7A | SalTrChr07Ag147830 |
| 82 | CYP72A1078_2A | SalTrChr02Ag047410 |
| 83 | CYP72A1078_2B | SalTrChr02Bg262860 |
| 84 | CYP72A1079_4A | SalTrChr04Ag080200 |
| 85 | CYP72A1079_4B | SalTrChr04Bg296020 |
| 86 | CYP72A1080_4A | SalTrChr04Ag080210 |
| 87 | CYP72A1081_4A | SalTrChr04Ag080260 |
| 88 | CYP72A1081_4B | SalTrChr04Bg296080 |
| 89 | CYP72A1083_6A | SalTrChr06Ag137130 |
| 90 | CYP72A1083_6B | SalTrChr06Bg356160 |
| 91 | CYP72A1084_7B | SalTrChr07Bg363290 |
| 92 | CYP72A1085_7A | SalTrChr07Ag148790 |
| 93 | CYP72A1085_7B | SalTrChr07Bg363120 |
| 94 | CYP72A1086_7A | SalTrChr07Ag148800 |
| 95 | CYP72A1087_7A | SalTrChr07Ag148810 |
| 96 | CYP72A1088_7A | SalTrChr07Ag148830 |
| 97 | CYP72A1088_7B | SalTrChr07Bg363140 |
| 98 | CYP72A1089_7A | SalTrChr07Ag153650 |
| 99 | CYP72A1089_7B | SalTrChr07Bg365920 |
| 100 | CYP72A1090_7A | SalTrChr07Ag153660 |
| 101 | CYP72A1090_7B | SalTrChr07Bg365940 |
| 102 | CYP72A1091_7A | SalTrChr07Ag153690 |
| 103 | CYP72A1091_7B | SalTrChr07Bg365900 |
| 104 | CYP72A1092_7B | SalTrChr07Bg365890 |
| 105 | CYP72A1093_7B | SalTrChr07Bg365840 |
| 106 | CYP72A1094_7A | SalTrChr07Ag153730 |
| 107 | CYP72A1094_7B | SalTrChr07Bg365830 |
| 108 | CYP72A1095_7B | SalTrChr07Bg365850 |
| 109 | CYP72A1096_7B | SalTrChr07Bg365860 |
| 110 | CYP72A1097_7B | SalTrChr07Bg365880 |
| 111 | CYP72D49_6A | SalTrChr06Ag142050 |
| 112 | CYP72D49_6B | SalTrChr06Bg348440 |
| 113 | CYP72Q1_7B | SalTrChr07Bg366140 |
| 114 | CYP73A272_3B | SalTrChr03Bg281980 |
| 115 | CYP73A273_8A | SalTrChr08Ag184900 |
| 116 | CYP74A218_3A | SalTrChr03Ag056060 |
| 117 | CYP74A218_3B | SalTrChr03Bg273040 |
| 118 | CYP74A228_9A | SalTrChr09Ag198520 |
| 119 | CYP74A228_9B | SalTrChr09Bg413110 |
| 120 | CYP74B64_6B | SalTrChr06Bg347730 |
| 121 | CYP75B159_1A | SalTrChr01Ag013240 |
| 122 | CYP75B159_1B | SalTrChr01Bg230340 |
| 123 | CYP75B215_3A | SalTrChr03Ag059720 |
| 124 | CYP75B216_1B | SalTrChr01Bg230350 |
| 125 | CYP76A145_1A | SalTrChr01Ag026900 |
| 126 | CYP76A145_1B | SalTrChr01Bg244060 |
| 127 | CYP76F237_7B | SalTrChr07Bg377200 |
| 128 | CYP76F238_7B | SalTrChr07Bg377190 |
| 129 | CYP76F239_6A | SalTrChr06Ag141240 |
| 130 | CYP76AD82_2A | SalTrChr02Ag030590 |
| 131 | CYP76AD82_2B | SalTrChr02Bg247780 |
| 132 | CYP76AD83a_2A | SalTrChr02Ag030770 |
| 133 | CYP76AD83b_2A | SalTrChr02Ag030780 |
| 134 | CYP76AD83c_2A | SalTrChr02Ag030790 |
| 135 | CYP76AD83_2B | SalTrChr02Bg247660 |
| 136 | CYP76AD84_2A | SalTrChr02Ag030800 |
| 137 | CYP76AD84_2B | SalTrChr02Bg247550 |
| 138 | CYP76AD85_3A | SalTrChr03Ag065470 |
| 139 | CYP76AD85_3B | SalTrChr03Bg277140 |
| 140 | CYP76AD86_3A | SalTrChr03Ag065490 |
| 141 | CYP76AD86_3B | SalTrChr03Bg277180 |
| 142 | CYP76AD87_5A | SalTrChr05Ag121380 |
| 143 | CYP76AD88_6A | SalTrChr06Ag137410 |
| 144 | CYP76AD89a_9B | SalTrChr09Bg426940a |
| 145 | CYP76AD89e_9A | SalTrChr09Ag204110 |
| 146 | CYP76AD90_9A | SalTrChr09Ag204120 |
| 147 | CYP76AD91_9B | SalTrChr09Bg427550 |
| 148 | CYP76AD91_9B | SalTrChr09Bg426960 |
| 149 | CYP76AD92a_9A | SalTrChr09Ag204140 |
| 150 | CYP76AD93_9A | SalTrChr09Ag216240 |
| 151 | CYP76AD93_9B | SalTrChr09Bg432040 |
| 152 | CYP76BN5_7B | SalTrChr07Bg379000 |
| 153 | CYP77A109_4A | SalTrChr04Ag083550 |
| 154 | CYP77B53_6B | SalTrChr06Bg347040 |
| 155 | CYP77B86_6B | SalTrChr06Bg358960 |
| 156 | CYP78A579_1A | SalTrChr01Ag012690 |
| 157 | CYP78A386_2A | SalTrChr02Ag044450 |
| 158 | CYP78A390_6A | SalTrChr06Ag143320 |
| 159 | CYP78A390_6B | SalTrChr06Bg359200 |
| 160 | CYP78A580_7A | SalTrChr07Ag161120 |
| 161 | CYP78A580_7B | SalTrChr07Bg380980 |
| 162 | CYP78A581_9A | SalTrChr09Ag215050 |
| 163 | CYP78A581_9B | SalTrChr09Bg430300 |
| 164 | CYP81B269_3A | SalTrChr03Ag054590 |
| 165 | CYP81B269_3B | SalTrChr03Bg271700 |
| 166 | CYP81B144_3A | SalTrChr03Ag054620 |
| 167 | CYP81B144_3B | SalTrChr03Bg271680 |
| 168 | CYP81BG54_6A | SalTrChr06Ag135690 |
| 169 | CYP81BG54_6B | SalTrChr06Bg351640 |
| 170 | CYP81BG55_6B | SalTrChr06Bg351670 |
| 171 | CYP81BG56_6B | SalTrChr06Bg351680 |
| 172 | CYP81CJ4_5B | SalTrChr05Bg319660 |
| 173 | CYP81CL3_3A | SalTrChr03Ag055610 |
| 174 | CYP81CR1_2A | SalTrChr02Ag048430 |
| 175 | CYP81CR1_2B | SalTrChr02Bg261750 |
| 176 | CYP81CS4_3B | SalTrChr03Bg269230 |
| 177 | CYP81CS5_3B | SalTrChr03Bg269240 |
| 178 | CYP81CT1_3A | SalTrChr03Ag065350 |
| 179 | CYP81CT1_3B | SalTrChr03Bg276980 |
| 180 | CYP81CT4_3B | SalTrChr03Bg277010 |
| 181 | CYP81DF1_2A | SalTrChr02Ag048620 |
| 182 | CYP81DF1_2B | SalTrChr02Bg261420 |
| 183 | CYP81DG1_7A | SalTrChr07Ag162660 |
| 184 | CYP81DG1_7B | SalTrChr07Bg376630 |
| 185 | CYP82D418_4B | SalTrChr04Bg303240 |
| 186 | CYP82D419_5B | SalTrChr05Bg330260 |
| 187 | CYP82D420_5B | SalTrChr05Bg330270 |
| 188 | CYP82D421a_6A | SalTrChr06Ag137610 |
| 189 | CYP82D421b_6A | SalTrChr06Ag137800 |
| 190 | CYP82D421_6B | SalTrChr06Bg356490 |
| 191 | CYP82D422_6A | SalTrChr06Ag137750 |
| 192 | CYP82D423_6A | SalTrChr06Ag137760 |
| 193 | CYP82D424_6A | SalTrChr06Ag137790 |
| 194 | CYP82D424_6B | SalTrChr06Bg356500 |
| 195 | CYP82D425_6B | SalTrChr06Bg356400 |
| 196 | CYP82D425_7B | SalTrChr07Bg376010 |
| 197 | CYP82D426_6B | SalTrChr06Bg356530 |
| 198 | CYP82D427_6B | SalTrChr06Bg356580 |
| 199 | CYP82U20_8A | SalTrChr08Ag191340 |
| 200 | CYP82U20_8B | SalTrChr08Bg396850 |
| 201 | CYP82BJ1_1A | SalTrChr01Ag017780 |
| 202 | CYP82BJ2_2B | SalTrChr02Bg258420 |
| 203 | CYP85A1_3A | SalTrChr03Ag070370 |
| 204 | CYP86A300_7A | SalTrChr07Ag167430 |
| 205 | CYP86A212_9A | SalTrChr09Ag202490 |
| 206 | CYP86B119_8A | SalTrChr08Ag168250 |
| 207 | CYP86B119_8B | SalTrChr08Bg383250 |
| 208 | CYP86B120_8A | SalTrChr08Ag168260 |
| 209 | CYP86B120_8B | SalTrChr08Bg383260 |
| 210 | CYP86C24_2A | SalTrChr02Ag045710 |
| 211 | CYP86C24_2B | SalTrChr02Bg257990 |
| 212 | CYP86C42_2A | SalTrChr02Ag045720 |
| 213 | CYP86C42_2B | SalTrChr02Bg257980 |
| 214 | CYP87A84_7A | SalTrChr07Ag161620 |
| 215 | CYP87A84_7B | SalTrChr07Bg381470 |
| 216 | CYP87D46_2A | SalTrChr02Ag029580 |
| 217 | CYP87D46_2B | SalTrChr02Bg245130 |
| 218 | CYP88A188_1A | SalTrChr01Ag023000 |
| 219 | CYP88A129_9A | SalTrChr09Ag206780 |
| 220 | CYP88A129_9B | SalTrChr09Bg421540 |
| 221 | CYP88A130_9A | SalTrChr09Ag206770 |
| 222 | CYP88A130_9B | SalTrChr09Bg421550 |
| 223 | CYP88A131_7A | SalTrChr07Ag167200 |
| 224 | CYP88A131_7B | SalTrChr07Bg382110 |
| 225 | CYP89A258_6A | SalTrChr06Ag125200 |
| 226 | CYP89A258_6B | SalTrChr06Bg339930 |
| 227 | CYP90A81_3A | SalTrChr03Ag066790 |
| 228 | CYP90A81_3B | SalTrChr03Bg287320 |
| 229 | CYP90B78_1A | SalTrChr01Ag022270 |
| 230 | CYP90B78_1B | SalTrChr01Bg237510 |
| 231 | CYP90C44_1A | SalTrChr01Ag022540 |
| 232 | CYP90C44_1B | SalTrChr01Bg226320 |
| 233 | CYP90D66_8A | SalTrChr08Ag184110 |
| 234 | CYP90D66_8B | SalTrChr08Bg396460 |
| 235 | CYP92A205_8A | SalTrChr08Ag173870 |
| 236 | CYP92A205_8B | SalTrChr08Bg389000 |
| 237 | CYP92A287_8B | SalTrChr08Bg395520 |
| 238 | CYP94A163_4A | SalTrChr04Ag075980 |
| 239 | CYP94A164_4B | SalTrChr04Bg292210 |
| 240 | CYP94B108_1A | SalTrChr01Ag010380 |
| 241 | CYP94B108_1B | SalTrChr01Bg227350 |
| 242 | CYP94C146_8A | SalTrChr08Ag184380 |
| 243 | CYP94C146_8B | SalTrChr08Bg406940 |
| 244 | CYP94C147_1A | SalTrChr01Ag014650 |
| 245 | CYP94C147_1B | SalTrChr01Bg231750 |
| 246 | CYP94D216_5A | SalTrChr05Ag102370 |
| 247 | CYP94D160_5A | SalTrChr05Ag102380 |
| 248 | CYP96A210_1A | SalTrChr01Ag020960 |
| 249 | CYP96A210_1B | SalTrChr01Bg235960 |
| 250 | CYP96A212_1A | SalTrChr01Ag021000 |
| 251 | CYP96A212_1B | SalTrChr01Bg236000 |
| 252 | CYP96A322_5A | SalTrChr05Ag118910 |
| 253 | CYP96A323_6B | SalTrChr06Bg348710 |
| 254 | CYP96A324_6B | SalTrChr06Bg354560 |
| 255 | CYP96A325_7A | SalTrChr07Ag165390 |
| 256 | CYP96A325_7B | SalTrChr07Bg374010 |
| 257 | CYP96A326_8B | SalTrChr08Bg391300 |
| 258 | CYP96A327_8B | SalTrChr08Bg397240 |
| 259 | CYP96A328_8B | SalTrChr08Bg397300 |
| 260 | CYP96A329_9B | SalTrChr09Bg428690 |
| 261 | CYP97A125_4A | SalTrChr04Ag094550 |
| 262 | CYP97A125_4B | SalTrChr04Bg307650 |
| 263 | CYP97B179_9A | SalTrChr09Ag194080 |
| 264 | CYP97B179_9B | SalTrChr09Bg409580 |
| 265 | CYP97C124_1A | SalTrChr01Ag022850 |
| 266 | CYP97C124_1B | SalTrChr01Bg238060 |
| 267 | CYP98A210_4B | SalTrChr04Bg309430 |
| 268 | CYP98A211_5A | SalTrChr05Ag101460 |
| 269 | CYP98A211_5B | SalTrChr05Bg317000 |
| 270 | CYP98A212_5A | SalTrChr05Ag101490 |
| 271 | CYP98A212_5B | SalTrChr05Bg317010 |
| 272 | CYP98A213_5A | SalTrChr05Ag101580 |
| 273 | CYP98A150_9A | SalTrChr09Ag193840 |
| 274 | CYP98A150_9B | SalTrChr09Bg409240 |
| 275 | CYP701A102_5A | SalTrChr05Ag099750 |
| 276 | CYP701A102_5B | SalTrChr05Bg318740 |
| 277 | CYP703A94_9B | SalTrChr09Bg430710 |
| 278 | CYP704A236_9A | SalTrChr09Ag204270 |
| 279 | CYP704A236_9B | SalTrChr09Bg427650 |
| 280 | CYP704A333_9A | SalTrChr09Ag204290 |
| 281 | CYP704A334_9B | SalTrChr09Bg427460 |
| 282 | CYP704B56_2A | SalTrChr02Ag042760 |
| 283 | CYP704B56_2B | SalTrChr02Bg267160 |
| 284 | CYP706C94_7A | SalTrChr07Ag164030 |
| 285 | CYP706C94_7B | SalTrChr07Bg375360 |
| 286 | CYP706Z6_7A | SalTrChr07Ag164020 |
| 287 | CYP706Z6_7B | SalTrChr07Bg375370 |
| 288 | CYP706Z7_7B | SalTrChr07Bg375380 |
| 289 | CYP707A241_7A | SalTrChr07Ag162960 |
| 290 | CYP707A241_7B | SalTrChr07Bg376470 |
| 291 | CYP707A378_8A | SalTrChr08Ag192790 |
| 292 | CYP707A378_8B | SalTrChr08Bg408160 |
| 293 | CYP710A131_9A | SalTrChr09Ag212860 |
| 294 | CYP710A131_9B | SalTrChr09Bg418080 |
| 295 | CYP711A193_3B | SalTrChr03Bg288480 |
| 296 | CYP712D43_3A | SalTrChr03Ag071720 |
| 297 | CYP712D43_3B | SalTrChr03Bg288190 |
| 298 | CYP714A47_9A | SalTrChr09Ag194040 |
| 299 | CYP714A47_9B | SalTrChr09Bg409510 |
| 300 | CYP714E65_9B | SalTrChr09Bg421130 |
| 301 | CYP715A72_5A | SalTrChr05Ag118210 |
| 302 | CYP715A72_5B | SalTrChr05Bg326050 |
| 303 | CYP716A406_3A | SalTrChr03Ag063600 |
| 304 | CYP716A406_3B | SalTrChr03Bg282150 |
| 305 | CYP716A407a_8A | SalTrChr08Ag178800 |
| 306 | CYP716A407b_8A | SalTrChr08Ag178840 |
| 307 | CYP716A407c_8A | SalTrChr08Ag178880 |
| 308 | CYP716A107d_8A | SalTrChr08Ag178920 |
| 309 | CYP716A407_8B | SalTrChr08Bg402330 |
| 310 | CYP716A408_8A | SalTrChr08Ag178930 |
| 311 | CYP716CL1_6B | SalTrChr06Bg356130 |
| 312 | CYP716CL2_6A | SalTrChr06Ag137370 |
| 313 | CYP718A32_8A | SalTrChr08Ag173770 |
| 314 | CYP718A32_8B | SalTrChr08Bg388830 |
| 315 | CYP721A119_3A | SalTrChr03Ag056890 |
| 316 | CYP721A119_3B | SalTrChr03Bg273920 |
| 317 | CYP722A10_9A | SalTrChr09Ag207330 |
| 318 | CYP722A10_9B | SalTrChr09Bg422190 |
| 319 | CYP734A147_1A | SalTrChr01Ag006790 |
| 320 | CYP734A147_1B | SalTrChr01Bg224050 |
| 321 | CYP734A147_00 | SalTrChr00g433110 |
| 322 | CYP735A107_2A | SalTrChr02Ag039930 |
| 323 | CYP735A107_2B | SalTrChr02Bg265210 |
| 324 | CYP735A108_3A | SalTrChr03Ag069280 |
| 325 | CYP735A108_3B | SalTrChr03Bg284900 |
| 326 | CYP736T1_4A | SalTrChr04Ag082200 |
| 327 | CYP736T1_4B | SalTrChr04Bg297630 |
| 328 | CYP736T2P_7B | SalTrChr07Bg370490 |
